# Analysis of the Cell Type-Dependence on the Arenavirus Z-Mediated Virus-Like Particle Production

**DOI:** 10.3389/fmicb.2020.562814

**Published:** 2020-09-30

**Authors:** Patrick I. Mpingabo, Shuzo Urata, Jiro Yasuda

**Affiliations:** ^1^Department of Emerging Infectious Diseases, Institute of Tropical Medicine, Nagasaki University, Nagasaki, Japan; ^2^Program for Nurturing Global Leaders in Tropical and Emerging Communicable Diseases, Graduate School of Biomedical Sciences, Nagasaki University, Nagasaki, Japan; ^3^National Research Center for the Control and Prevention of Infectious Diseases, Nagasaki University, Nagasaki, Japan

**Keywords:** arenavirus, L-domain, Z, cell type-dependence, virus-like particle

## Abstract

Several arenaviruses are highly pathogenic to humans, causing hemorrhagic fever. Discovery of anti-arenavirus drug candidates is urgently needed, although the molecular basis of the host- and organ-specific pathogenicity remains to be fully elucidated. The arenavirus Z protein facilitates production of virus-like particles (VLPs), providing an established method to assess virus budding. In this study, we examined the efficiency of VLP production by solely expressing Z protein of several different arenaviruses. In addition, we analyzed the role of the late (L)-domain of the arenavirus Z protein, which is essential for the interaction with ESCRT proteins, in VLP production among different cell lines. VLP assay was performed using Z proteins of Junín virus (JUNV), Machupo virus (MACV), Tacaribe virus (TCRV), Latino virus (LATV), Pichinde virus (PICV), and Lassa virus (LASV) in six different cell lines: HEK293T, Huh-7, A549, Vero76, BHK-21, and NIH3T3 cells. JUNV, MACV, and LASV Z proteins efficiently produced VLPs in all tested cell lines, while the efficiencies of VLP production by the other arenavirus Z proteins were cell type-dependent. The contribution of the L-domain(s) within Z protein to VLP production also highly depended on the cell type. These results suggested that each arenavirus has its own particle-production mechanism, which is different among the cell types.

## Introduction

Several arenaviruses including Lassa, Lujo, Junín, Machupo, Guanarito, Sabia, and Chapare viruses (LASV, LUJV, JUNV, MACV, GTOV, SABV, and CHPV) cause severe symptoms such as hemorrhagic fever. There is currently no vaccine or drug approved by the Food and Drug Administration (FDA) (Buchmeier et al., 2007). Most arenaviruses infect humans through direct contacts with body fluids of infected humans or animals, bites by infected rodents, or aerosol inhalation of their excretions. Arenaviruses are classified into two groups: Old World (OW) arenaviruses including LASV and LUJV; and New World (NW) arenaviruses including JUNV, MACV, GTOV, SABV, and CHPV (Charrel et al., 2008). Distribution of arenaviruses is closely related to that of the natural reservoir, rodents. Due to an increase in international travel over the past few decades, a potential risk for the worldwide spread of arenavirus has attracted a lot of attention from public health organizations (McCormick et al., 1986; Ericsson et al., 2001).

The virus budding/release process is one of ideal antiviral targets. Oseltamivir and zanamivir are well-known anti-influenza viral drugs, which target the viral neuraminidase activity in digesting the interaction between the viral GP (hemagglutinin) and the host cell sialic acid to release progeny virions from the cell surface (Palese, 2007; Ryu, 2016). However, there is currently no antiviral drug that targets the virus budding/release of highly virulent viruses, including arenaviruses.

**FIGURE 1:**
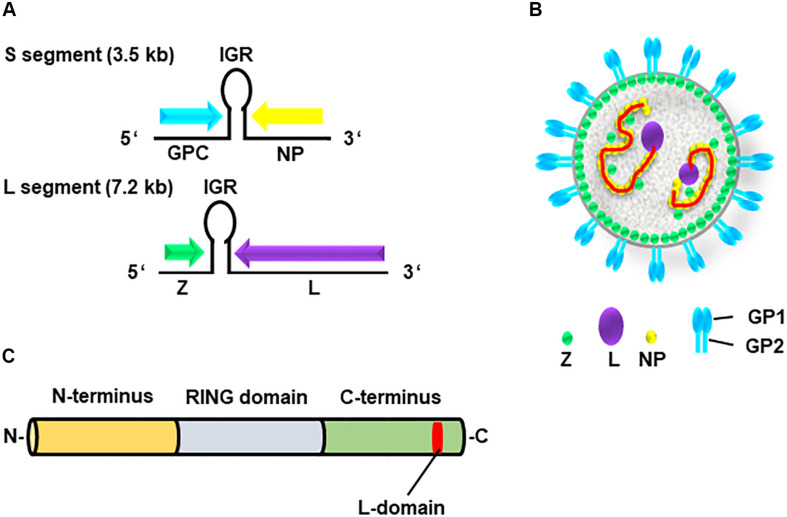
Genome and structure of arenavirus. **(A)** The genome of arenaviruses consists of two segments of single-stranded ambisense RNA, S and L. Each segment encodes two viral proteins separated by an intergenic region (IGR), a non-coding region forming a hairpin structure. The S segment encodes viral nucleoprotein (NP) and glycoprotein precursor (GPC), whereas the L segment encodes viral RNA-dependent RNA polymerase (L) and the matrix protein (Z). **(B)** Structure of the arenavirus: GP1/2 (blue), L (purple), NP (yellow) and Z (green). The viral genome (red) is encapsidated by NP. **(C)** Z protein consists of three domains, N-terminus (orange), central RING domain (light blue) and C-terminus (bright green) containing the L-domain (red).

The genome of arenavirus comprises two ambisense single-stranded RNA segments, S and L (Figures 1A,B). Each segment encodes two viral proteins: [1] the S segment encodes the GP precursor and the nucleoprotein (NP); while the [2] L segment encodes the RNA-dependent RNA polymerase (L) and matrix protein (Z) (Urata et al., 2006; Urata and de la Torre, 2011; Urata and Yasuda, 2012). The arenavirus Z protein has multiple functions in the viral life cycle through interactions with host cellular proteins (Capul et al., 2007; Schlie et al., 2010; Fehling et al., 2012). This viral protein consists of three domains, the N-terminal domain, the central RING domain, and the C-terminal L-domain (Figure 1C) (Perez et al., 2003, 2004; Gamsjaeger et al., 2007).

Most enveloped viruses, including filoviruses, retroviruses and arenaviruses, possess viral matrix proteins that play a central role in viral assembly, budding, and release. Most of the viral matrix proteins encode short amino acid motifs called late (L)-domains that interact with a component of the ESCRT complex, and play a critical role at the late step of viral budding (Yasuda and Hunter, 1998; Martin-Serrano et al., 2003; Wolff et al., 2013). Three amino acid motifs, PT/SAP, PPXY and YPXnL (or YXXL), have been well established as L-domains (Bieniasz, 2006); these motifs have been shown to interact with the host factors Tsg101, Nedd4-like E3 ubiquitin ligases and ALIX/AIP1, respectively (Garrus et al., 2001; Martin-Serrano et al., 2001; Yasuda et al., 2002).

Arenavirus Z proteins possess a variety of L-domains, and their role in virus budding have previously been examined (Strecker et al., 2003; Wang et al., 2012). Z proteins of OW arenaviruses contain both PT/SAP (or similar to PT/SAP) and PPPY motifs, except for LUJV whose L-domain contains only the PSAP motif. In contrast, Z proteins of most NW arenaviruses possess only PT/SAP motifs, although both the PICV and the White water arroyo virus (WWAV) possess overlapping sequences of PT/SAP and PPPY-like motifs (APPY), known as PT/SAPPY, which are similar to those of the Ebola virus VP40 (PTAPPPY) (Harty et al., 2000; Urata and Yasuda, 2012). Although the TCRV Z protein only contains the ASAP sequence, which is similar to PT/SAP, this specific ASAP sequence does not appear to function as an L-domain (Urata et al., 2009; Groseth et al., 2010).

The progeny virion production of several enveloped viruses, which is regulated by L-domains, has been reported to be cell type-dependent. For example, membrane targeting of the Gag proteins of HIV-1 (Ono and Freed, 2004), HTLV-1 (Dorweiler et al., 2006), and M-PMV (Narahara and Yasuda, 2015) are regulated in a cell type-dependent manner. Knowledge of the cell type-dependence on arenavirus infection will provide insights into virological basis in each host and organ, leading to a discovery of key cellular factors/pathways and of candidates for anti-viral targets. However, the cell type-dependence on the budding process of arenaviruses has not been fully clarified. In this study, we examined the cell type-dependence of the budding process using Z proteins of six arenaviruses, including functions of L-domains, in six different cell lines.

## Materials and Methods

### Cell Lines, Plasmids and Antibodies

**FIGURE 2:**
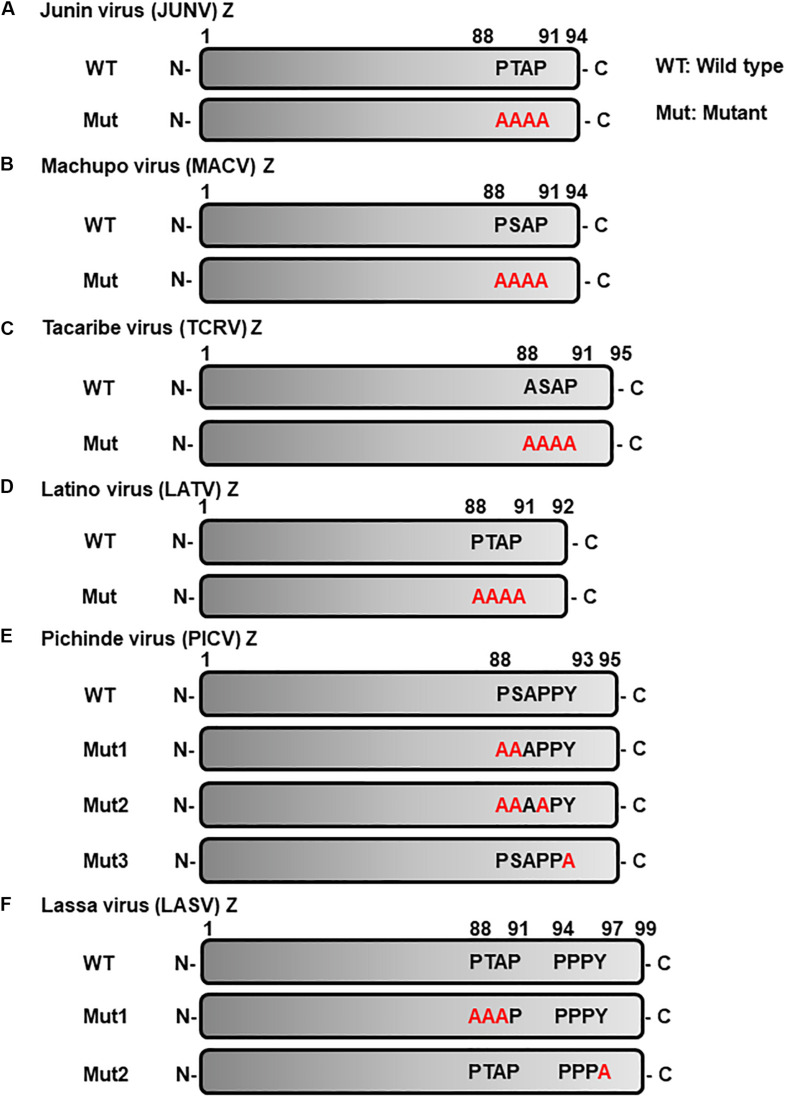
Schematic representation of L-domain within arenavirus Z proteins and the L-domain mutants used in this study. L-domains within Z proteins of the following arenaviruses. **(A)** Junín virus (JUNV), **(B)** Machupo virus (MACV), **(C)** Tacaribe virus (TCRV), **(D)** Latino virus (LATV), **(E)** Pichinde virus (PICV), **(F)** Lassa virus (LASV), and their respective alanine mutant forms. WT, wild-type; Mut, mutant.

HEK293T (Human embryonic kidney epithelial cell), Huh-7 (Human hepatocarcinoma epithelial cell), A549 (Human lung epithelial cancer cell), Vero76 (African green monkey kidney epithelial cell), BHK-21 (Baby hamster kidney fibroblast cell), and NIH3T3 (Mouse fibroblast cell) were selected based on the fact that epithelial cells form body barriers and play a pivotal role not only in initial virus infection but also in the process of virus progeny release during arenavirus infection (Dylla et al., 2008; Schlie et al., 2010). In addition, NIH3T3 cells were derived from the mouse that represents the natural reservoir of arenaviruses (Buchmeier et al., 2007); whereas, Vero76 cells were derived from the monkey that is a potential animal model for arenavirus research (Kenyon et al., 1992). These cell lines were cultured in Dulbecco’s Modified Eagle Medium (DMEM) (Thermo Fischer Scientific, United States) supplemented with 10% fetal bovine serum and 1% penicillin/streptomycin (Thermo Fischer Scientific). The Z proteins of both pathogenic and non-pathogenic arenavirus strains were selected according to their genetic classification (OW, LASV; NW clade A, PICV; NW clade B, JUNV, MACV and TCRV; NW clade C, LATV) (Charrel et al., 2008; Urata and Yasuda, 2012). The pCAGGS plasmids expressing wild-type (WT) or mutant (Mut) Z proteins of the above listed arenaviruses, which possess C-terminal FLAG (for JUNV, MACV, TCRV, LATV, PICV, and LASV) or C-terminal HA (for TCRV) tags (Niwa et al., 1991; Urata et al., 2009), were kindly provided by Dr. J.C. de la Torre (The Scripps Research Institute). The LASV Z expression plasmid was previously described (Urata et al., 2006; Urata and Yasuda, 2015). To construct expression plasmids for the L-domain mutants (Figure 2), KOD plus mutagenesis kit (Toyobo, Japan) was used according to the manufacturer’s protocol using the following primer sets: JUNV Z-Mut, 5′-GTACCGGTGGAGGCAGCAGCAGCACCACCAGG-3′ and 5′-TGTGATTGTGGTGGGCAGGGGC-3′; MACV Z-Mut, 5′-GGAGGCAGCTGCCGCCCCACCAGGAGGAG-3′ and 5′-ACGGGAACTGTGATGGATGTCGG-3′; LATV Z-Mut, 5′-CATAACTGCAGCAGCAGCGCAACTCAACGGAGG-3′ and 5′-CCGACTTCAATGTAGGTTGGAATTG-3′; PICV Z-Mut1, 5′-GTTTCTGGAGAGTGCGGCTGCAGCTCCCTATG-3′ and 5′-CATAGGGAGGTGCAGCCGCACTCTCCAGAAAC-3′; PICV Z-Mut2, 5′-GTTTCTGGAGAGTGCGGCTGCACCTCCC TATG-3′ and 5′-CATAGGGAGGTGCAGCCGCACTCTCCAGA AAC-3′; PICV Z-Mut3, 5′-GTCTGCACCTCCCGCTGAGC CAGGAGGAG-3′ and 5′-CTCCTCCTGGCTCAGCGGGAGGT GCAGAC-3′. The following antibodies: anti-FLAG polyclonal antibody (Sigma, United States), anti-HA polyclonal antibody (QED Bioscience, United States), anti-LASV Z polyclonal antibody (Urata et al., 2006), and mouse anti-β-actin monoclonal antibody (Sigma), were used as primary antibodies. Anti-rabbit IgG (Promega, United States) and anti-mouse IgG (Sigma), which are conjugated with horseradish peroxidase (HRP), were used as secondary antibodies.

### Virus-Like Particle (VLP) Assay

Virus-like particle assays were performed as previously described (Urata et al., 2006, 2009). Briefly, transfection of Z-WT or -Mut expression plasmids was performed using Trans-IT LT-1 (Mirus Bio, United States) for HEK293T, Huh-7, and BHK-21 cells; and Lipofectamine 3000 (Invitrogen, United States) for A549, Vero76 and NIH3T3 cells. At 48 or 72 h post-transfection (hpt), VLPs, released into culture media, were collected and pelleted through a 20% sucrose cushion by ultracentrifugation (195,600 × *g*, 30 min, 4°C). Cells were lysed with Lysis A buffer (1 M Tris-HCl (pH 8.0), 0.5 M EDTA, 1% NP-40, and 0.4% Na-deoxycholate) and cleared by centrifugation (13,000 × *g*, 5 min, 4°C). Both VLPs and cell lysates were subjected to SDS-PAGE and subsequently analyzed by western blot (WB). Relative VLP production was calculated from band intensities as a VLP/Cell ratio, which was normalized for JUNV Z or WT and set as 1.0.

### SDS-PAGE and Western Blot (WB)

Virus-like particle and cell lysate samples were loaded on a 15% SDS polyacrylamide gel followed by transfer to a nitrocellulose blotting membrane (Amersham, Germany). Membranes were blocked for 1 h in 5% non-fat dry milk (Blocking buffer), then incubated with the corresponding primary antibody for 2 h at room temperature. After washing with wash buffer (0.2 M Tris-HCl (pH 7.5), 8.76 g/L NaCl and 0.25% Tween 20), the membranes were incubated with the appropriate HRP-conjugated secondary antibodies for 2 h at room temperature. Membranes were washed with wash buffer three times for 10 min each and subsequently imaged on an image analyzer LAS-3000 (Fujifilm, Japan) using ECL prime chemiluminescent reagent (GE Healthcare, Italy) according to the manufacturer’s instructions. The representative data of immunoblots repeated at least four times are shown in each figure.

### Statistical Analyses and Quantification

Statistical analyses were performed using GraphPad Prism 6 software, and the one-sample Student’s *t*-test (column statistics) was used to compare WT and Mut. The following *p*-values: ^∗^*p* < 0.05; ^∗∗^*p* < 0.01; ^∗∗∗^*p* < 0.001, were considered statistically significant. In all of the graphs, data are shown as the mean and standard deviation of four independent experiments.

## Results

### Z-Mediated VLP Production of Arenaviruses in Six Different Cell Lines

**FIGURE 3:**
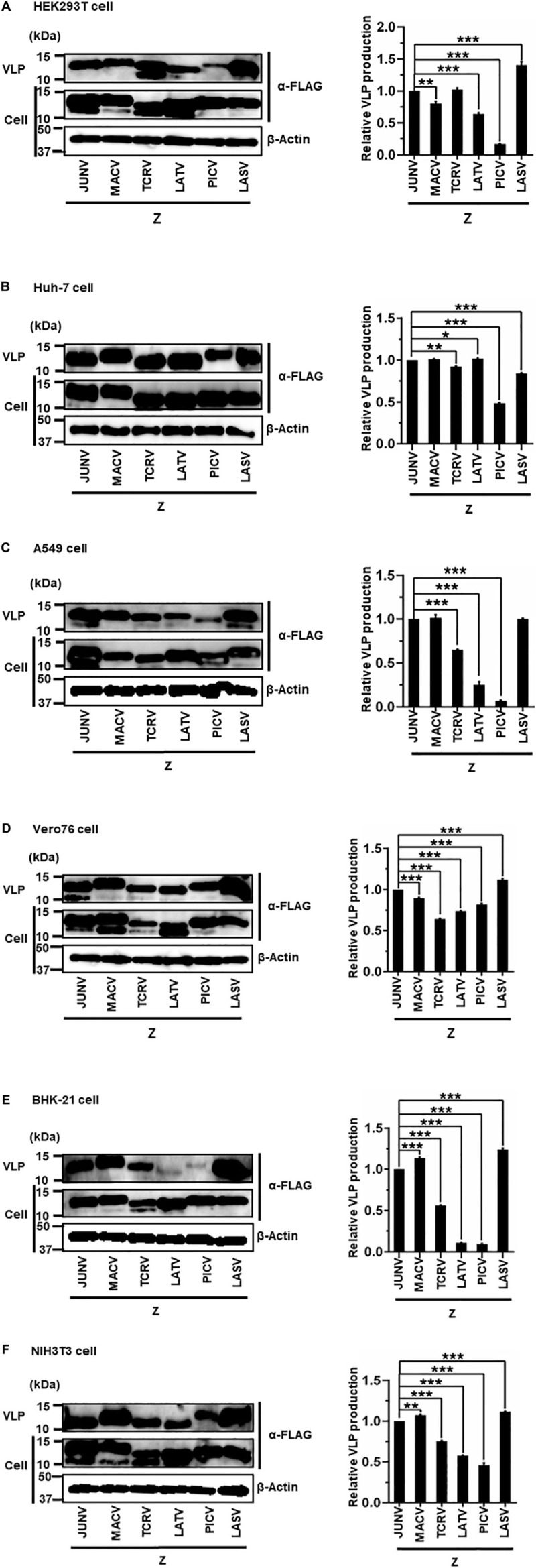
Arenavirus Z-mediated VLP production. HEK293T cells **(A)**, Huh-7 cells **(B)**, A549 cells **(C)**, Vero76 cells **(D)**, BHK-21 cells **(E)**, and NIH3T3 cells **(F)** were transfected with expression plasmids for JUNV, MACV, TCRV, LATV, PICV, or LASV Z-FLAG. At 48 h post-transfection (hpt) **(A–C,E)** or 72 hpt **(D,F)**, VLPs and whole-cell lysates were collected and analyzed by western blot (WB). In all experiments, actin served as a loading control. VLP production by JUNV Z-WT was set at 1.0 as a standard, and the data shown are averages and standard deviations of four independent experiments (right panels). *p < 0.05; **p < 0.01; ***p < 0.001.

The sole expression of arenavirus Z proteins can induce VLP production in cells (Perez et al., 2003; Strecker et al., 2003; Urata et al., 2006). First, we examined whether Z proteins of JUNV, MACV, TCRV, LATV, PICV, and LASV can produce and release VLPs from cells. In this study, we used six cell lines from different origins, HEK293T, Huh-7, A549, Vero76, BHK-21, and NIH3T3 cells. At 48 or 76 hpt of Z expression plasmids, culture supernatants containing VLPs and cell lysates were prepared and analyzed by WB to detect FLAG-tagged Z proteins using an anti-FLAG antibody. As shown in Figure 3 and Table 1, JUNV, MACV, and LASV Z efficiently produced VLPs in all six cell lines. PICV Z produced significantly lower amounts of VLP in HEK293T (17%), A549 (7%), and BHK-21 (9%) cells, and slightly lower amounts of VLP in Huh-7 (49%) and NIH3T3 (46%) cells, compared to JUNV Z. The ratios of TCRV Z-mediated VLP production in A549, Vero76 and BHK-21 cells were 64%, 64%, and 56%, respectively, when compared to JUNV Z. LATV Z expression efficiently produced VLP, relative to JUNV Z expression in Huh-7 and Vero76 cells, while those in HEK293T and NIH3T3 cells were slightly lower relative to JUNV Z (64% and 56%, respectively). VLP production in LATV Z A549 and BHK-21 cells was significantly lower relative to JUNV Z (25% and 11%, respectively). Taken together, these data show that the efficiency of Z-mediated VLP production of TCRV, PICV, and LATV is cell-type dependent.

**TABLE 1:** The efficiency of arenavirus Z-mediated VLP production.

Arenavirus Z WT	HEK293T % (SD)	Huh-7 % (SD)	A549 % (SD)	Vero76 % (SD)	BHK-21 % (SD)	NIH3T3 % (SD)
JUNV	100 (0)	100 (0)	100 (0)	100 (0)	100 (0)	100 (0)
MACV	80 (3.5)**	101 (0.7)	101 (3.8)	89 (1)***	114 (1.4)***	107 (1.2)**
TCRV	102 (2.5)	92 (0.8)**	64 (3.4)***	64 (0.7)***	56 (0.6)***	75 (0.8)***
LATV	64 (1.9)***	102 (0.7)*	25 (3.4)***	74 (0.7)***	11 (0.8)***	56 (0.6)***
PICV	17 (0.5)***	49 (0.8)***	7 (0.9)***	82 (1.4)***	9 (0.8)***	46 (2.5)***
LASV	140 (5.8)***	84 (0.8)***	100 (0.9)	112 (1.5)***	124 (1.5)***	111 (0.7)***

VLP production by JUNV was at 100% as standard. WT, wild-type; JUNV, Junín virus; MACV, Machupo virus; TCRV, Tacaribe virus; LATV, Latino virus; PICV, Pichinde virus; LASV, Lassa virus; WT, wild-type; SD, standard deviation; *p < 0.05, **p < 0.01, ***p < 0.001.

### Cell-Type Dependent Functions of L-Domains in VLP Production

We next examined if the L-domains within Z proteins of arenaviruses function in a cell-type dependent manner. WT and L-domain Muts of each arenavirus Z protein were expressed in six different cell lines, and VLP production levels were compared between WT and the different Muts.

#### (i) Role of the PTAP Motif in JUNV Z-Mediated VLP Production

**FIGURE 4:**
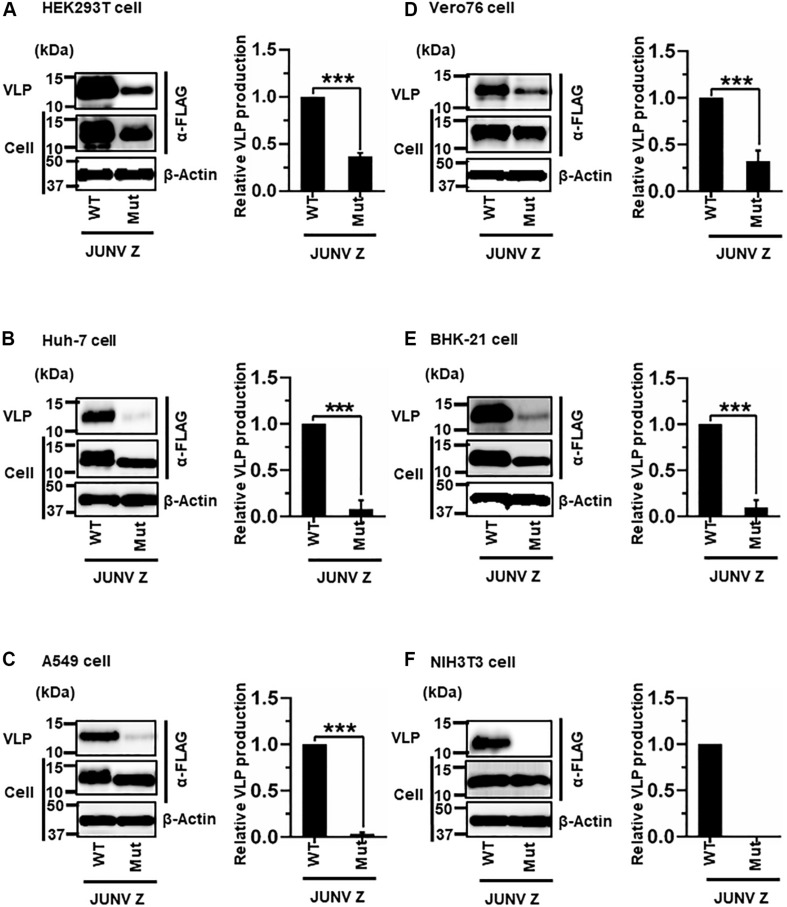
Role of the PTAP motif in JUNV Z-mediated VLP production: **(A)** HEK293T cells were transfected with the expression plasmid for WT (PTAP) or mutant (Mut; PTAP → AAAA) JUNV Z-FLAG using TransIT LT1 transfection reagent. At 48 hpt, VLPs and whole-cell lysates were collected and analyzed by western blot (WB). **(B)** Huh-7 cells were transfected and analyzed as described above. **(C)** A549 cells were transfected with the same expression plasmids using Lipofectamine 3000 transfection reagent and analyzed as described in panel **(A)**. **(D)** Vero76 cells were transfected with the same expression plasmids as described in panel **(A)**. At 72 hpt, VLPs and whole-cell lysates were collected and analyzed by WB. **(E)** BHK-21 cells were transfected and analyzed as described in panel **(A)**. **(F)** NIH3T3 cells were transfected and analyzed as described in panel **(D)**. In all performed experiments, actin served as a loading control. VLP production from JUNV Z-WT was set at 1.0 as a standard, and the data shown are averages and standard deviations of four independent experiments (right panels). WT, wild-type; Mut, mutant. ***p < 0.001.

JUNV Z possesses a PTAP motif in its C-terminus, in the form of an L-domain (Figure 2A) (Urata and de la Torre, 2011; Urata and Yasuda, 2012). To examine whether this PTAP motif plays a role in VLP production, cells were transfected with either WT or Mut (PTAP → AAAA) Z expression plasmids (Figure 2A). VLPs and cell lysates were analyzed by WB. In all cell lines examined in this study, Mut showed an apparent reduction in VLP production efficiency, although the intracellular expression levels of Z-Mut in HEK293T, Huh-7, Vero76, and BHK-21 cells were only slightly lower relative to those of WT (Figure 4). Specifically, reduced expression levels were significant in Huh-7, A549, and BHK-21 cells (92%, 97%, and 91%, respectively) (Table 2 and Figures 4B,C,E). VLP production was completely abolished in NIH3T3 cells expressing the Mut protein (Figure 4F). These results clearly indicate that the PTAP motif plays a critical role in JUNV Z-mediated VLP production, and the degree of importance of the L-domain is dependent on cell type.

**TABLE 2:** The contribution of late (L)-domain on arenavirus Z-mediated VLP production.

Arenavirus	HEK293T % (SD)	Huh-7 % (SD)	A549 % (SD)	Vero76 % (SD)	BHK-21 % (SD)	NIH3T3 % (SD)
**JUNV Z**						
WT	100 (0)	100 (0)	100 (0)	100 (0)	100 (0)	100 (0)
Mut	37 (3.9)***	8 (9.5)***	3 (1.8)***	32 (11.5)***	9 (8.4)***	0 (0)
**MACV Z**						
WT	100 (0)	100 (0)	100 (0)	100 (0)	100 (0)	100 (0)
Mut	21 (21.3)**	12 (23.9)**	11 (13.7)***	42 (6.4)**	42 (19.7)**	0 (0)
**TCRV Z**						
WT	100 (0)	100 (0)	100 (0)	100 (0)	100 (0)	100 (0)
Mut	88 (10.4)	24 (8.1)***	71 (15.4)	81 (12.6)	19 (2.3)***	96 (2.7)
**LATV Z**						
WT	100 (0)	100 (0)	100 (0)	100 (0)	100 (0)	100 (0)
Mut	12 (1.6)***	88 (9.6)	94 (12.5)	107 (8.7)	108 (9)	99 (3.6)
**PICV Z**						
WT	100 (0)	100 (0)	100 (0)	100 (0)	100 (0)	100 (0)
Mut1	8 (0.7)***	103 (3)	9 (0.4)***	126 (7.8)	8 (0.8)***	49 (0.7)***
Mut2	6 (1.1)***	6 (1.5)***	9 (0.7)***	81 (8.5)	8 (1.1)***	7 (0.5)***
Mut3	10 (0.3)***	50 (2.1)***	7 (0.4)***	92 (9.7)	43 (5.5)***	50 (1.3)***
**LASV Z**						
WT	100 (0)	100 (0)	100 (0)	100 (0)	100 (0)	100 (0)
Mut1	96 (5)	42 (0.9)***	5 (0.3)***	44 (1.6)***	17 (0.5)***	14 (1.6)***
Mut2	27 (1)***	6 (0.1)***	4 (0.3)***	27 (2.1)***	0 (0)	16 (1.1)***

VLP production by WT was at 100% as standard. JUNV, Junín virus; MACV, Machupo virus; TCRV, Tacaribe virus; LATV, Latino virus; PICV, Pichinde virus; LASV, Lassa virus; WT, wild-type; Mut, mutant; SD, standard deviation; **p < 0.01, ***p < 0.001.

#### (ii) Role of the PSAP Motif in MACV Z-Mediated VLP Production

**FIGURE 5:**
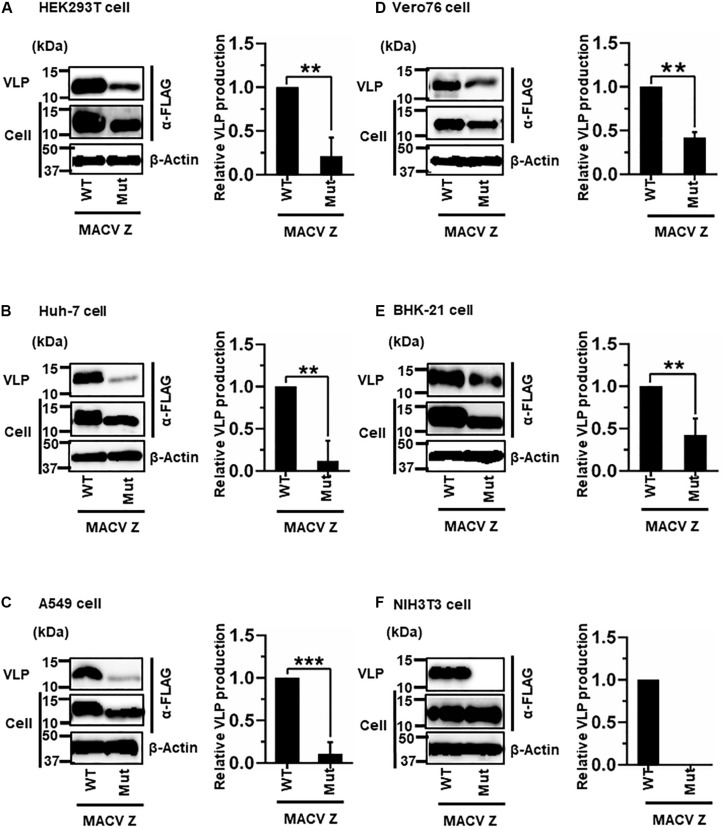
Role of the PSAP motif in MACV Z-mediated VLP production: **(A)** HEK293T cells were transfected with the expression plasmid for the WT (PSAP) or mutant (Mut; PSAP → AAAA) MACV Z-FLAG using TransIT LT1 transfection reagent. At 48 hpt, VLPs and cell lysates were collected and analyzed by western blot (WB). **(B)** Huh-7 cells were transfected and analyzed as described above. **(C)** A549 cells were transfected with the same expression plasmids using Lipofectamine 3000 transfection reagent and analyzed as described in panel **(A)**. **(D)** Vero76 cells were transfected with the same expression plasmids as described in panel **(A)**. At 72 hpt, VLPs and whole-cell lysates were collected and analyzed by WB. **(E)** BHK-21 cells were transfected and analyzed as described in panel **(A)**. **(F)** NIH3T3 cells were transfected and analyzed as described in panel **(D)**. In all performed experiments, actin served as a loading control. VLP production from MACV Z-WT was set at 1.0 as a standard, and the data shown are averages and standard deviations of four independent experiments (right panels). WT, wild-type; Mut, mutant. **p < 0.01, ***p < 0.001.

MACV Z contains a PSAP motif in its C-terminus (Figure 2B) (Urata and de la Torre, 2011; Urata and Yasuda, 2012). We examined the role of this PSAP motif in VLP production using six cell lines. Similar the JUNV results shown in Figure 4, intracellular expression levels of Z-Mut in HEK293T, Huh-7, A549, Vero76, and BHK-21 cells were slightly lower than those of WT (Figures 5A–E). The efficiency of VLP production by MACV Z-Mut (PSAP → AAAA) was much lower than that of WT across all cell lines (Figure 5). Specifically, in HEK293T, Huh-7, and A549 cells, reduction in VLP production by Mut was significant (79%, 88%, and 89%, relative to WT, respectively) (Table 2 and Figures 5A–C). In Vero76 and BHK-21 cells, the reduction in VLP production was also observed, but to a much modest degree (58% reduction for both) (Table 2 and Figures 5D,E). VLP production was completely abolished in NIH3T3 cells expressing the Mut protein (Figure 5F). These results indicate that the PSAP motif plays an important role in MACV Z-mediated VLP production, and the degree to which the L-domain contributes to the role of the MACV Z protein, in VLP production, is also dependent on cell type, as observed in JUNV.

#### (iii) Role of the ASAP Sequence in TCRV Z-Mediated VLP Production

**FIGURE 6:**
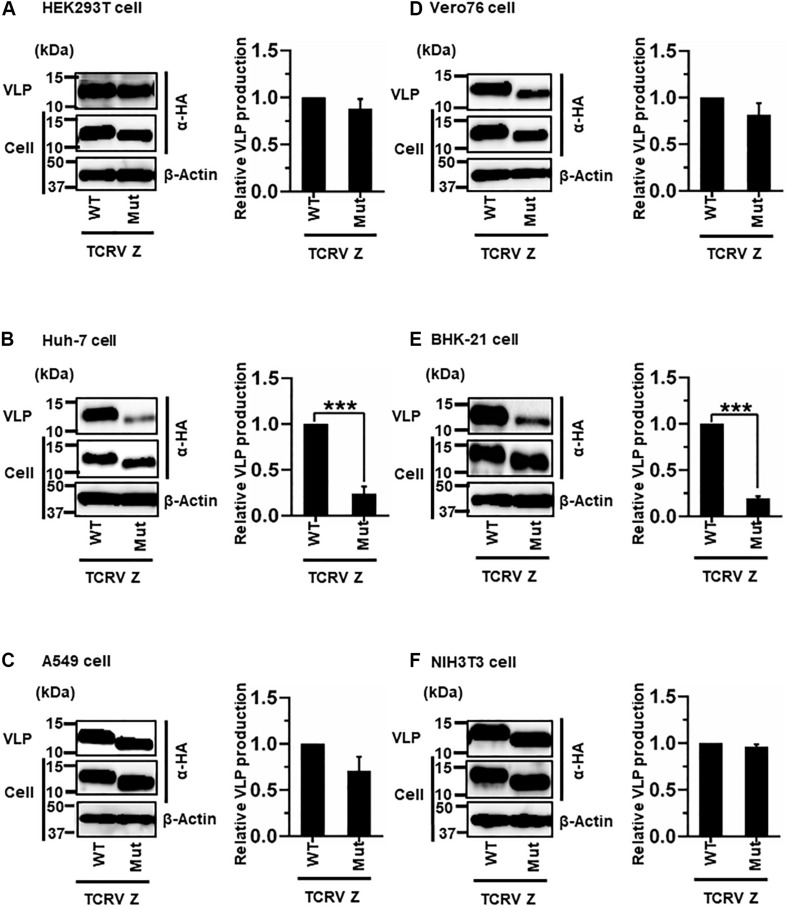
Role of the ASAP motif in TCRV Z-mediated VLP production: **(A)** HEK293T cells were transfected with the expression plasmid for the WT (ASAP) or mutant (Mut; ASAP → AAAA) TCRV Z-HA using TransIT LT1 transfection reagent. At 48 hpt, VLPs and cell lysates were collected and analyzed by western blot (WB). **(B)** Huh-7 cells were transfected and analyzed as described above. **(C)** A549 cells were transfected with the same expression plasmids using Lipofectamine 3000 transfection reagent and analyzed as described in panel **(A)**. **(D)** Vero76 cells were transfected with the same expression plasmids as described in panel **(A)**. At 72 hpt, VLPs and whole-cell lysates were collected and analyzed by WB. **(E)** BHK-21 cells were transfected and analyzed as described in panel **(A)**. **(F)** NIH3T3 cells were transfected and analyzed as described in panel **(D)**. In all performed experiments, actin served as a loading control. VLP production from TCRV Z-WT was set at 1.0 as a standard, and the data shown are averages and standard deviations of four independent experiments (right panels). WT, wild-type; Mut, mutant. ***p < 0.001.

Most NW arenavirus Z proteins possess a PT/SAP motif, in the form of an L-domain at their C-terminus. However, TCRV Z only possesses an ASAP motif, similar to PT/SAP, at its C-terminus (Figure 2C). Previous studies have shown that the ASAP sequence does not contribute to Z-mediated VLP production in 293T cells (Urata et al., 2009; Groseth et al., 2010). For a better understanding of this observation, we further analyzed the role of ASAP on VLP production in different cell lines. WT and Mut (ASAP → AAAA) Z, with a C-terminal HA tag, were detected using an anti-HA antibody. TCRV Z-Mut exhibited only a slight reduction in VLP production compared to Z-WT in HEK293T, A549, Vero76, and NIH3T3 cells (12%, 29%, 19%, and 4% reduction, respectively) (Figures 6A,C,D,F and Table 2). In contrast, VLP production mediated by Z-Mut was significantly reduced compared to that by Z-WT in Huh-7 and BHK-21 cells (76% and 81% reduction, respectively) (Figures 6B,E and Table 2). These results strongly suggest that the ASAP sequence, within TCRV Z, functions as an L-domain in some cell lines.

#### (iv) Role of the PTAP Motif in LATV Z-Mediated VLP Production

**FIGURE 7:**
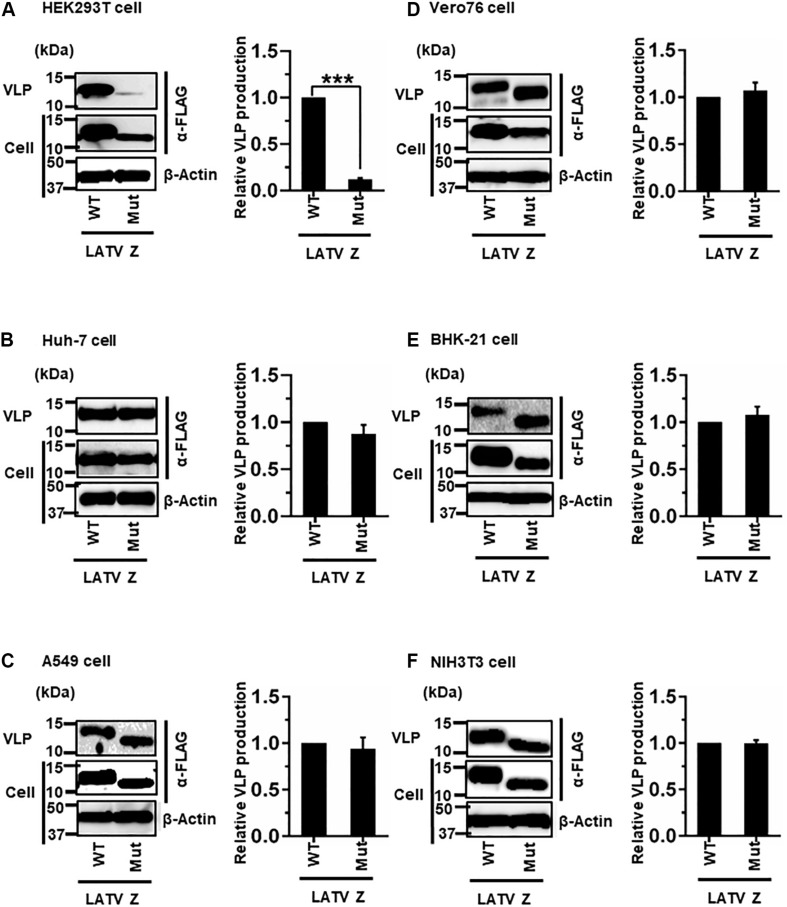
Role of the PTAP motif in LATV Z-mediated VLP production: **(A)** HEK293T cells were transfected with the expression plasmid for the WT (PTAP) or mutant (Mut; PTAP → AAAA) LATV Z-FLAG using TransIT LT1 transfection reagent. At 48 hpt, VLPs and cell lysates were collected and analyzed by western blot (WB). **(B)** Huh-7 cells were transfected and analyzed as described above. **(C)** A549 cells were transfected with the same expression plasmids using Lipofectamine 3000 transfection reagent and analyzed as described in panel **(A)**. **(D)** Vero76 cells were transfected with the same expression plasmids as described in panel **(A)**. At 72 hpt, VLPs and whole-cell lysate were collected and analyzed by WB. **(E)** BHK-21 cells were transfected and analyzed as described in panel **(A)**. **(F)** NIH3T3 cells were transfected and analyzed as described in panel **(D)**. In all performed experiments, the actin served as a loading control. VLP production from LATV Z-WT was set at 1.0 as a standard, and the data shown are averages and standard deviations of four independent experiments (right panels). WT, wild-type; Mut, mutant. ***p < 0.001.

LATV Z possesses a PTAP motif in its C-terminus (Figure 2D). As shown in Figure 7, efficient VLP production, mediated by Z-Mut (PTAP → AAAA), was observed in most cell lines, although intracellular expression of Z-Mut was lower than that of WT in all six cell lines (Figures 7B–F). Only in HEK293T cells, the efficiency of Z-Mut-mediated VLP production was much lower than that of WT-mediated production (12%) (Figure 7A), suggesting that the LATV Z PTAP motif functions as an L-domain only in HEK293T cells, but not in Huh-7, A549, Vero76, BHK-21, and NIH3T3 cells. It should be noted that VLP production in A549 and BHK-21 cells was much lower than that in the other cell lines, as seen in Table 1 and Figures 3C,E (Table 2 and Figures 7C,E).

#### (v) Role of the PSAPPY Motif in PICV Z-Mediated VLP Production

**FIGURE 8:**
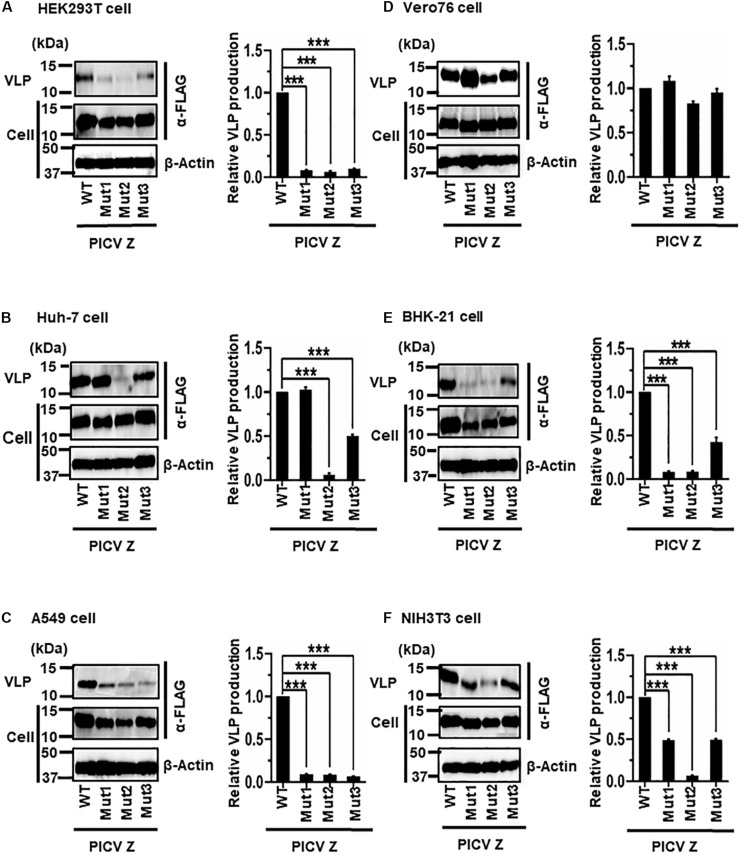
Role of the PSAPPY motif in PICV Z-mediated VLP production: **(A)** HEK293T cells were transfected with the expression plasmid for the WT (PSAPPY) or three mutants, PICV Z-Mut1 (PSAPPY → AAAAPY), PICV Z-Mut2 (PSAPPY → AAAPPY) and PICV Z-Mut3 (PSAPPY → PSAPPA), using TransIT LT1 transfection reagent. At 48 hpt, VLPs and cell lysates were collected and analyzed by western blot (WB). **(B)** Huh-7 cells were transfected and analyzed as described above. **(C)** A549 cells were transfected with the same expression plasmids using Lipofectamine 3000 transfection reagent and analyzed as described in panel **(A)**. **(D)** Vero76 cells were transfected with the same expression plasmids as described in panel **(A)**. At 72 hpt, VLPs and whole-cell lysates were collected and analyzed by WB. **(E)** BHK-21 cells were transfected and analyzed as described in panel **(A)**. **(F)** NIH3T3 cells were transfected and analyzed as described in panel **(D)**. In all performed experiments, actin served as a loading control. VLP production from PICV Z-WT was set at 1.0 as a standard, and the data shown are averages and standard deviations of four independent experiments (right panels). WT, wild-type; Mut, mutant. ***p < 0.001.

PICV Z possesses an overlapping PSAPPY motif at its C-terminus, which is involved in either the production of virions in BHK-21 cell line clones (BSR) or VLP production in HEK293T cells (Figure 2E) (Strecker et al., 2003; Urata and Yasuda, 2012). In order to better understand the contribution of the PSAPPY motifs to PICV Z-mediated VLP production, we examined the importance of the PSAPPY motif in the production of VLPs across six cell lines using three different mutants, Mut1 (PSAPPY → AAAAPY), Mut2 (PSAPPY → AAAPPY), and Mut3 (PSAPPY → PSAPPA) (Figure 2E). VLP production by all three mutants in HEK293T and A549 cells was much lower than that of WT (Figures 8A,C), while all three mutants in Vero76 cells had levels comparable to WT (Figure 8D). In Huh-7 cells, while Mut1 had VLP levels comparable to WT, Mut2 and Mut3 displayed a decrease in VLP production (Figure 8B). In BHK-21 and NIH3T3 cells, VLP production by all three mutants was lower than that by WT at different levels (Table 2 and Figures 8E,F).

#### (vi) Role of the PTAP and PPPY Motifs in LASV Z-Mediated VLP Production

**FIGURE 9:**
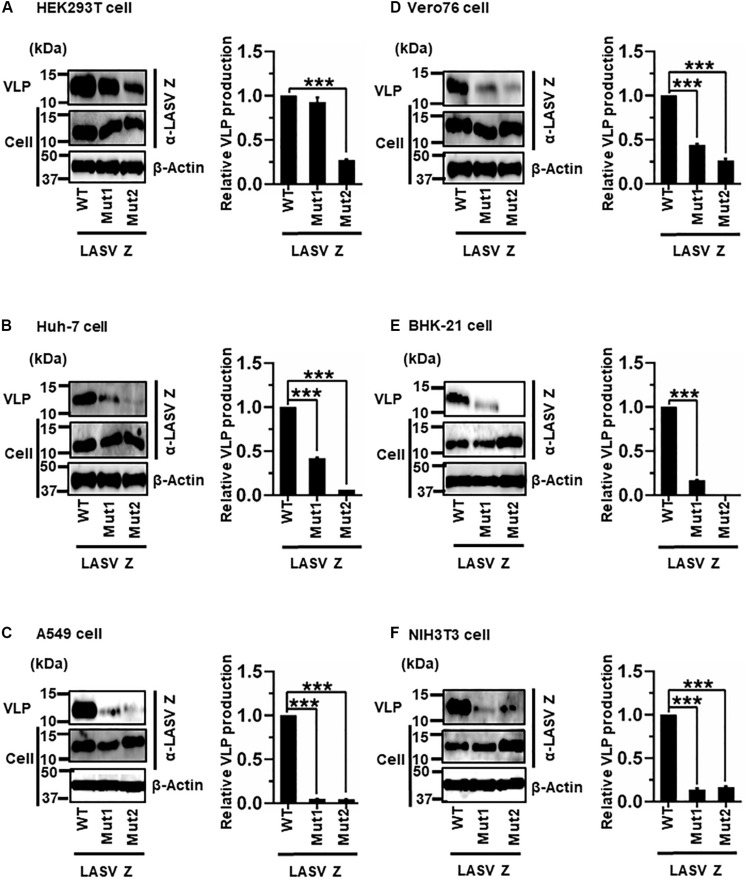
Role of the PTAP and PPPY motifs in LASV Z-mediated VLP production: **(A)** HEK293T cells were transfected with the expression plasmid for the WT (PTAP-PPPY), Mut1 (PTAP → AAAP), or Mut2 (PPPY → PPPA) LASV Z using TransIT LT1 transfection reagent. At 48 hpt, VLPs and cell lysates were collected and analyzed by western blot (WB). **(B)** Huh-7 cells were transfected and analyzed as described above. **(C)** A549 cells were transfected with the same expression plasmids using Lipofectamine 3000 transfection reagent and analyzed as described in panel **(A)**. **(D)** Vero76 cells were transfected with the same expression plasmids as described in panel **(A)**. At 72 hpt, VLPs and whole-cell lysates were collected and analyzed by WB. **(E)** BHK-21 cells were transfected and analyzed as described in panel **(A)**. **(F)** NIH3T3 cells were transfected and analyzed as described in panel **(D)**. In all performed experiments, actin served as a loading control. VLP production from LASV Z-WT was set at 1.0 as a standard, and the data shown are averages and standard deviations of four independent experiments (right panels) WT, wild-type; Mut, mutant. ***p < 0.001.

LASV Z possesses two complete L-domains, PTAP and PPPY, at its C-terminus (Figure 2F) (Perez et al., 2003; Strecker et al., 2003; Urata et al., 2006). To examine, in detail, the role of PTAP and PPPY in LASV Z-mediated VLP production, VLP production mediated by two Z mutants, Mut1 (PTAP → AAAP) and Mut2 (PPPY → PPPA) (Figure 2F), was compared to that by WT in the six cell lines. Although Mut1 had VLP levels comparable to WT in HEK293T cells (Figure 9A), the Mut1 level was more substantially reduced in the other cell lines (Figures 9B–F). Contrarily, Mut2-mediated VLP production was much lower relative to that of WT in all six cell lines (Table 2 and Figures 9A–F). Taken together, the data suggest that the PPPY motif functions as an L-domain and plays a critical role in VLP production and that the PTAP motif functions as an L-domain in a cell-type dependent manner.

## Discussion

The arenavirus Z protein has been shown to regulate virus budding via its C-terminal L-domain(s) (Perez et al., 2003; Urata et al., 2006; Urata and Yasuda, 2012). To date, cell type-dependence of arenavirus Z-mediated VLP production and the role(s) of the L-domain in this process have not been examined.

Our analysis showed that JUNV, MACV, and LASV Z efficiently produced VLPs in all cell lines (Figure 3 and Table 1). Previous studies reported similar observations that JUNV and MACV Z efficiently produce VLPs in HEK293T and BHK-21 cell lines (Perez et al., 2003; Radoshitzky et al., 2011; Ziegler et al., 2018). These data indicated a common mechanism to produce VLPs in JUNV and MACV with the following reasons: they are genetically close to each other; and they possess typical L-domains. In contrast, TCRV, LATV, and PICV Z-mediated VLP production dramatically changed in each cell line. These observations clearly demonstrate a cell type-dependence of the Z-mediated VLP production of each arenavirus, possibly indicating the differences of associating host factors in the VLP production process. Contrary to the result in this study, a previous report showed a sufficient VLP production by PICV Z in HEK293T cells (Wang et al., 2012), suggesting that small differences in experimental settings could contribute to the amount of VLP production (e.g., VLP centrifugation method, transfection process, cell culture condition).

JUNV, MACV, and LATV Z proteins share the typical PT/SAP motif in the L-domain (Urata and de la Torre, 2011; Urata and Yasuda, 2012). Interestingly, the Z protein with L-domain mutation (Z-Mut) of JUNV and MACV remarkably reduced the production of VLPs in all tested cell lines, whereas LATV Z-Mut expression caused no or small changes in VLP production in most cell lines. The differences in the effect of Z-Mut would highlight the distinct host cell factors/pathways required for Z-mediated VLP production among these three arenaviruses, although it is still unclear how the Z protein except the L-domain functions to produce VLPs. TCRV Z possesses an L-domain-like motif, ASAP, instead of a typical PT/SAP motif. TCRV Z-Mut expression showed a unique result different from that of other arenaviruses, depicting a complexity in molecular mechanisms underlying cell type-dependent VLP production. A previous study could provide us a significant perspective for contribution of other viral proteins; NP protein enhances the efficiency of TCRV Z-mediated VLP production (Groseth et al., 2010). Our results with JUNV, MACV, and TCRV Z are in agreement with previous reports (Perez et al., 2003; Urata et al., 2009; Groseth et al., 2010), strengthening a reliability of the VLP assays in this study.

PICV Z contains the PSAPPY motif that consists of two overlapping typical motifs, PSAP and APPY. This overlapping PSAPPY motif is similar to the EBOV VP40 L-domain that has the ability to interact with cellular proteins in the budding or virus particle production process (Harty et al., 2000; Yasuda et al., 2003; Urata and Yasuda, 2012). Wang et al. (2012) demonstrated that the two amino acid substitutions (P88A/S89A, P91A/P92A, and P92A/Y93A) in the PSAPPY motif of PICV Z significantly affect viral growth although there is a minor effect on its self-budding activity. Interestingly, PICV Z mutants exhibited different VLP production levels in Huh-7 cell lines, indicating an important role of the amino acid P91 in VLP production in Huh-7 cells, although further analysis is required to fully understand the function of the PSAPPY motif in PICV Z.

LASV is an important OW arenavirus of public health concern due to its high pathogenicity to humans (McCormick et al., 1986). Only a few matrix proteins, including LASV Z, EBOV VP40, and M-PMV Gag, possess two typical motifs, PTAP and PPPY (Gottwein et al., 2003; Perez et al., 2003; Urata and Yasuda, 2012). Our results indicate that a PPPY motif, especially tyrosine (Y), has a critical role in VLP production with LASV Z in most cell lines. In contrast, the importance of the PTAP motif in VLP production was dependent on cell lines, indicating an interaction of the PTAP motif with cell type-specific cellular factors. The result in this study is consistent with a previous report using the M-PMV Gag (Narahara and Yasuda, 2015).

The N-terminal domain (NTD) of Z protein is also involved in the Z-mediated virus budding via myristoylation and membrane association (Perez et al., 2004; Strecker et al., 2006; Urata et al., 2009). In addition, NTD has another function in host immune response through the inhibition of retinoic acid-inducible gene 1 (RIG-I) like receptor (RLR)-dependent interferon release pathway and macrophage activation (Fan et al., 2010; Xing et al., 2015a, b), possibly indicating the relation between the Z-mediated VLP budding process and the immune response. Thus, further analysis would be needed to clarify whether NTD functions are also cell type-dependent or not.

In conclusion, we found that the L-domain in the arenavirus Z protein significantly contribute to VLP production in a cell type-dependent manner, suggesting that each arenavirus has different modes of VLP production. Further investigations are required to identify the cellular factors that interact with the L-domain for efficient VLP production in each cell line, and to elucidate the molecular mechanisms underlying the cell type-dependence of VLP production among a variety of arenaviruses. This study provides new insights into the mechanism of arenavirus-like particle production, and may provide a better understanding into their pathogenesis, thereby potentially leading to an effective antiviral strategy targeting the virus budding/release process from host cells.

## Data Availability Statement

The raw data supporting the conclusions of this article will be made available by the authors, without undue reservation.

## Author Contributions

SU designed the study. PM performed all experiments, analyzed data, and prepared the manuscript. JY supervised the study. SU and JY analyzed data and edited the manuscript. All authors contributed to the article and approved the submitted version.

## Conflict of Interest

The authors declare that the research was conducted in the absence of any commercial or financial relationships that could be construed as a potential conflict of interest.

## Abbreviations

EBOV: Ebola virus
ESCRT: endosomal sorting complex required for transport
GP: glycoprotein
HIV: human immunodeficiency virus
HTLV-1: human T-cell leukemia virus type 1
JUNV: Junín virus
L-domain: late-domain
LASV: Lassa virus
LATV: Latino virus
LF: Lassa fever
MACV: Machupo virus
M-PMV: Mason-Pfizer monkey virus
Mut: mutant
NP: nucleoprotein
PICV: Pichinde virus
PSV5: paramyxovirus simian virus 5
RING: really interesting new gene
SDS-PAGE: sodium dodecyl sulfate-polyacrylamide gel electrophoresis
TCRV: Tacaribe virus
TSG 101: tumor susceptible gene 101
VLP: virus-like particle
WT: wild-type.
